# Bite First, Bleed Later: How Philippine *Trimeresurus* Pit Viper Venoms Hijack Blood Clotting

**DOI:** 10.3390/toxins17040185

**Published:** 2025-04-07

**Authors:** Daniel Albert E. Castillo, Lorenzo Seneci, Abhinandan Chowdhury, Marilyn G. Rimando, Bryan G. Fry

**Affiliations:** 1 The Graduate School, University of Santo Tomas, España Boulevard, Manila 1008, Philippines or decastillo@ust.edu.ph (D.A.E.C.); mgrimando@ust.edu.ph (M.G.R.); 2 Adaptive Biotoxicology Lab, School of the Environment, University of Queensland, St Lucia 4072, Australia; l.seneci@uq.edu.au (L.S.); abhinandan.choudhury@uq.edu.au (A.C.); 3 Department of Biological Sciences, College of Science, University of Santo Tomas, España Boulevard, Manila 1008, Philippines; 4 Avilon Wildlife Conservation Foundation and School of Practical Veterinary Management, Inc., 9003 GP Sitio Gulod, Barangay San Isidro, Rodriguez 1860, Rizal, Philippines; 5 Research Center for the Natural and Applied Sciences, University of Santo Tomas, España Boulevard, Manila 1008, Philippines

**Keywords:** *Trimeresurus*, coagulotoxicity, antivenom, pseudo-procoagulant, fibrinogen

## Abstract

The Philippines has a high diversity of venomous snake species, but there is minimal information on their envenomation effects. This is evidenced by the small number of case reports, the poor reporting of envenomation cases, and the absence of specific antivenoms apart from one against the Philippine cobra (*Naja philippinensis*). This study sought to profile the action of selected Philippine pit viper venoms on blood coagulation and to investigate whether commercially available non-specific antivenoms can provide adequate protection against these venoms. Venom from the pit vipers *Trimeresurus flavomaculatus* and *Trimeresurus mcgregori* were subjected to coagulation assays, antivenom cross-neutralization tests, and thromboelastography. Venoms from both species were able to clot human plasma and isolated human fibrinogen. Consistent with pseudo-procoagulant/thrombin-like activity, the resulting fibrin clots were weak and transient, thereby contributing to net anticoagulation through the depletion of fibrinogen levels. Clotting factors fIXa and fXa were also inhibited by the venoms, further contributing to the net anticoagulant activity. Monovalent and polyvalent antivenoms from the Thai Red Cross Society were effective against both venoms, indicating cross-neutralization of venom toxins; the polyvalent antivenom was able to rescue fibrinogen clotting to a greater degree than the monovalent antivenom. Our findings highlight the coagulopathic effects of these pit viper venoms and suggest the utility of procuring the non-specific antivenoms for areas in the Philippines with a high risk for pit viper envenomation.

## 1. Introduction

Snake venom is a complex exophysiological secretion produced in a specialized gland [1]. It is composed of proteins, polypeptides, and other molecules, and is used for defense and feeding [2]. It evolved in snakes primarily—but not exclusively—as a chemical means to subdue prey [3]. Depending on the snake species, human envenomations cause varying degrees of injury ranging from local effects such as erythematous inflammation or tissue necrosis to systemic effects including hemorrhagic shock and neurotoxicity [4,5]. These effects are mainly brought about by toxins affecting physiological systems reachable by the bloodstream [6,7,8,9] and may cause long-term sequelae or mortality [10].

Most snakebites occur in tropical countries where venomous snakes are more commonly found, but the true global burden of snakebites is underestimated and underappreciated. Estimates suggest that about 5.5 million snakebites occur globally, with 1.8 million cases of envenomation and 94,000–138,000 deaths. Most cases are recorded in Asia and sub-Saharan Africa [11,12,13]. Because of the high disease burden, coupled with poor availability of antivenom in the most affected countries, the World Health Organization reinstated snakebite in 2017 as a neglected tropical disease requiring increased awareness and research [14,15].

In the Philippines, local neglect of snakebites is evident in the lack of up-to-date, verifiable epidemiological data. Around 40 species of venomous snakes can be found in the Philippines [16], but snakebite cases are grossly underreported and largely anecdotal [17,18]. Envenomation effects are also poorly documented, with sporadic case reports [19,20] among the few sources of information. In addition, none of these reports investigated the underlying toxic mechanisms of snake venom. This deficiency of available information on Philippine snake venoms may play a part in the general lethargy for antivenom development: to date, the only antivenom available in the country is the Philippine Cobra Antivenom (PCAV) made of horse immunoglobulins against the venom of the Philippine cobra (*Naja philippinensis*) [21,22,23].

Pit vipers (Viperidae: Crotalinae) are venomous snakes primarily distinguished by the presence of deep sensory pits located between the eyes and nostrils which are used for heat sensing, especially in low light [24]. Their venom is typically hemotoxic, although neurotoxicity is common in some species [9,25,26,27,28,29]. The Philippines has five species of pit vipers: *Trimeresurus flavomaculatus*, *Trimeresurus mcgregori*, *Trimeresurus schultzei*, *Tropidolaemus philippensis*, and *Tropidolaemus subannulatus* [30]. Three of these species (*T. flavomaculatus*, *T. philippensis*, and *T. subannulatus*) are listed under Category 2 of the WHO’s snake antivenom guidelines, indicating that they are medically important venomous snakes capable of causing morbidity and mortality, but data on their venom effects are insufficient [31]. On the other hand, because of their limited habitat range, *T. mcgregori* (Batanes Islands) and *T. schultzei* (Palawan) are not listed in the guidelines. Still, cases of snakebite by both species have been reported [20,32].

While there are numerous studies on the hemotoxic effects of pit vipers in general, there is still a gap in knowledge in relation to the hemotoxicity and sequelae of envenomation by Philippine species. Additionally, given the local lack of antivenom against pit viper bites, the generalized policy on snakebite management, and the reliance on alternative medicine particularly in rural areas [33,34], the true burden of snakebites from pit vipers has yet to be adequately measured. Antivenom development and first aid treatment have primarily focused on neurotoxic and fatal envenomations by the endemic cobra species [16,17,35,36], but this overlooks the possible morbidity of pit viper envenomation [25,37,38,39,40].

Thus, the present study tested the venom of *T. flavomaculatus* and *T. mcgregori* for coagulotoxic effects on human plasma and fibrinogen and whether they can inhibit key clotting factors. Moreover, considering the technically demanding process of antivenom production, we also tested non-specific antivenoms for cross-neutralization activities against these species. Our findings underscore the significant hemotoxic risks associated with envenomation by these endemic pit vipers, necessitating a re-evaluation of local snakebite treatment strategies. By testing possible cross-neutralizing antivenoms, we seek to propose improvements to current treatment guidelines for snakebite management. Local policymakers may explore the potential benefit of storing non-specific yet effective antivenoms in areas with a high risk of snakebite or consider developing specific antivenoms for these species.

## 2. Results

**Figure 1:**
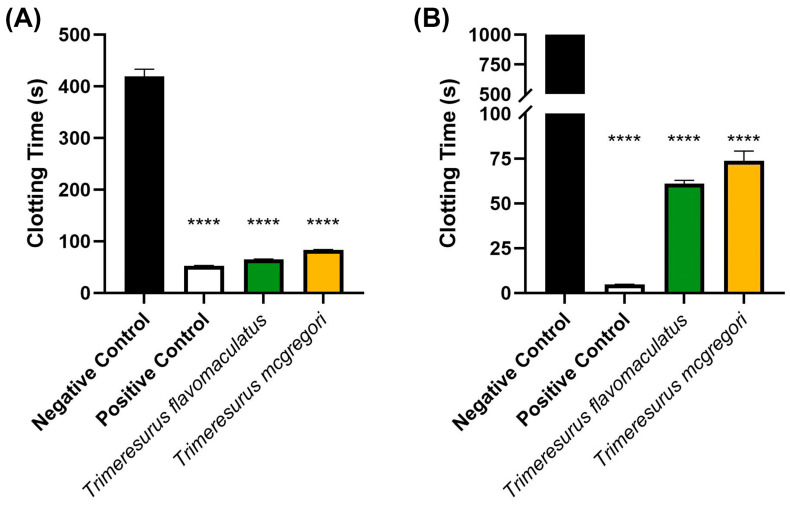
Initial 20 µg/mL venom concentration clotting analysis on human (**A**) plasma and (**B**) fibrinogen. Clotting time was measured until either plasma or fibrinogen clotted or the machine maximum observation period of 999 s was reached. Data are presented as mean ± SD, *n* = 3; asterisks (****) indicate *p* < 0.0001 when compared to negative control.

Initial coagulation analysis of the *Trimeresurus* venoms (20 µg/mL concentration) revealed that both species were able to clot human plasma faster (*p <* 0.0001) than a spontaneous clotting control (negative control). Venom from *T. flavomaculatus* clotted the plasma at 65.40 ± 0.36 s, while *T. mcgregori* clotted plasma at 83.20 s ± 0.83. The negative control clotting time was 419.3 ± 13.97 s (Figure 1A). A similar coagulation analysis was performed on human fibrinogen (Figure 1B), showing that both *T*. *flavomaculatus* and *T*. *mcgregori* were also able to clot fibrinogen significantly faster (*p* < 0.0001) than the negative control. *T*. *flavomaculatus* clotted fibrinogen at 60.88 ± 1.93 s. On the other hand, *T*. *mcgregori* was able to clot fibrinogen at 73.77 ± 5.45 s.

**Figure 2:**
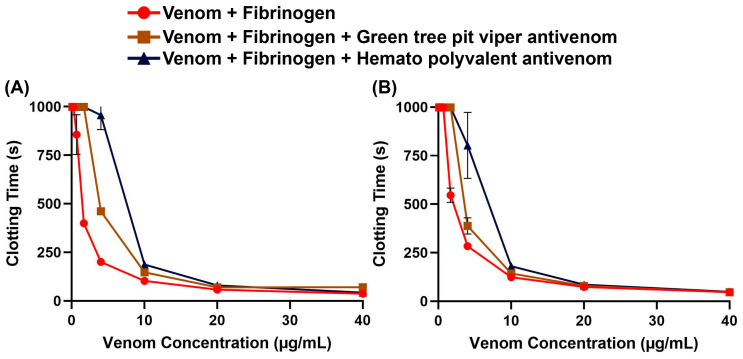
Antivenom effects on human fibrinogen clotting activity. Clotting time was measured until fibrinogen clotted or the maximum monitoring time of 999 s was reached. Data are presented as mean ± SD, *n* = 3; note that several error bars are absent because they are smaller than the icons. (**A**) *Trimeresurus flavomaculatus*; (**B**) *Trimeresurus mcgregori*.

To assess whether non-specific antivenoms are effective against the *Trimeresurus* venoms, we performed concentration–response curves on fibrinogen with and without antivenom. We found that both the Hemato Polyvalent Antivenom against viperids with known hemotoxic venom (*Daboia siamensis*, *Calloselasma rhodostoma*, and *Trimeresurus albolabris*) and the Green Tree Pit Viper Antivenom against *T. albolabris* were able to rescue fibrinogen clotting at varying concentrations of venom (Figure 2). Between the venom samples, *T*. *flavomaculatus* was neutralized at a wider range of concentrations (Table 1).

**Figure 3:**
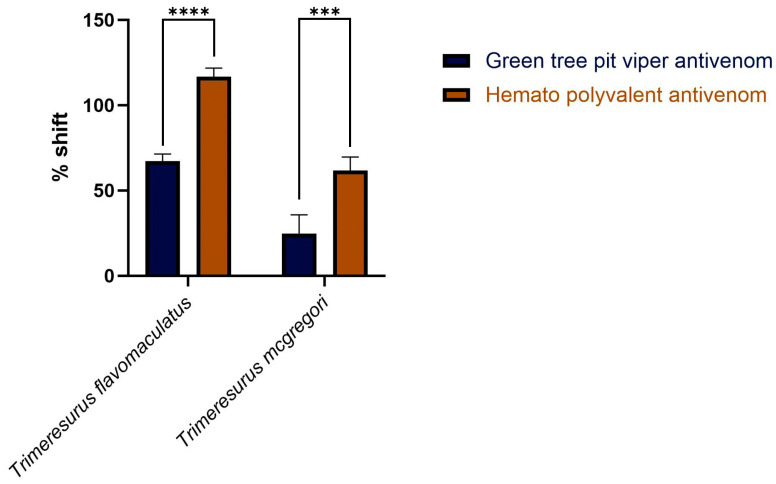
Percentage shift in the fibrinogen-clotting-time area under the curve in the presence of the antivenoms. This is calculated by the formula: [(AUC of venom + antivenom in fibrinogen/AUC of venom in fibrinogen) − 1] × 100. A percent shift value of 0 indicates no cross-neutralization, and a value greater than 0 indicates cross-neutralization by the antivenoms. Blue bars: Green Tree Pit Viper Antivenom; brown bars: Hemato Polyvalent Antivenom. Data are presented as mean ± SD, *n* = 3; asterisks indicate *** *p* = 0.0001, **** *p* < 0.0001.

Area under the curve (AUC) calculations revealed that both the monovalent Green Tree Pit Viper Antivenom and the Hemato Polyvalent Antivenom yielded percentage shifts > 0, indicating cross-neutralization of toxins from *T. flavomaculatus* and *T. mcgregori*. Notably, the Hemato Polyvalent Antivenom gave a significantly higher percentage shift than its monovalent counterpart in both samples (Figure 3).

Thromboelastography was performed to quantify the venoms’ effects on clot strength in plasma and fibrinogen [41]. Three parameters were measured for this study: the split point (SP), which is the time at which the tracings show a divergence into two arms, representing the initial formation of the fibrin clot [42]; amplitude (A), which is the width of the diverged arms at the end of the tracing, representing clot strength; and reaction time (R), which is the time taken until the formation of a detectable clot where the A first reaches a width of 2 mm [43].

**Figure 4:**
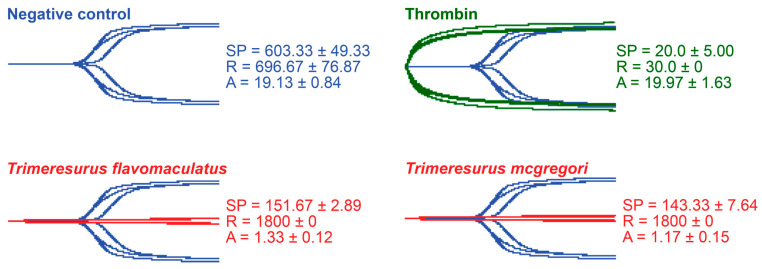
Thromboelastography with human plasma. Blue tracings = spontaneous clotting control (negative control); green tracings = thrombin control; red tracings = venom samples. All traces are overlaid with negative control. SP = split point, i.e., the time in seconds until the start of clot formation. R = reaction time, which is the time in seconds until an observable clot (i.e., A ≥ 2 mm) is formed. A = amplitude, which is the width in mm of the tracing at the end of the observation time, representing clot strength. Parameters are *n* = 3 and are presented as mean ± SD. Total run time is 1800 s.

While venoms from both species caused plasma clotting earlier than the negative control, neither venom sample was able to generate a clot where A ≥ 2 mm throughout the 30 min run time. The venom-induced clots were weaker, consistent with a pseudo-procoagulant/thrombin-like mechanism of action (Figure 4).

**Figure 5:**
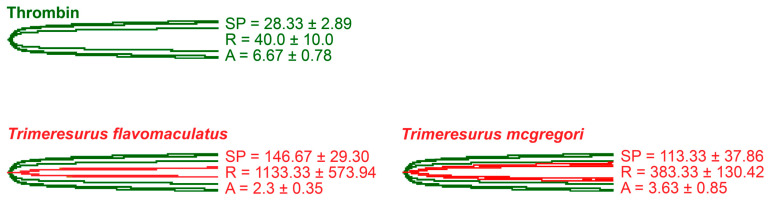
Thromboelastography with fibrinogen. Green tracings = thrombin control; red tracings = venom samples. All traces are overlaid with thrombin control. SP = split point, i.e., the time in seconds until the start of clot formation. R = reaction time, which is the time in seconds until an observable clot (i.e., A ≥ 2 mm) is formed. A = amplitude, which is the width in mm of the tracing at the end of the observation time, representing clot strength. Parameters are *n* = 3 and are presented as mean ± SD. Total run time is 1800 s.

As thrombin is the clotting cascade serine protease that cleaves fibrinogen to form fibrin [44], it was used as the positive control for thromboelastography on fibrinogen (Figure 5). Against fibrinogen, thrombin generated an SP of 28.33 ± 2.89 s, an R of 40.0 ± 10.0 s, and an A of 6.67 ± 0.78 mm. The parameters measured in the presence of thrombin were significantly different from those measured in the venom samples. Split point comparisons between the thrombin control and the venoms revealed that thrombin clotted fibrinogen faster than all samples (*p* = 0.0009 for *T*. *flavomaculatus*; *p* = 0.0072 for *T*. *mcgregori*) and generated an observable clot (R) faster than the *T*. *flavomaculatus* venom (*p* = 0.0050 *T*. *flavomaculatus*). As for *T*. *mcgregori*, owing to the relatively high standard deviation, its R values are not significantly different (*p* = 0.4017) from thrombin, but the individual values are consistently higher, indicating a slower rate of fibrinogenolysis. Most importantly, thrombin cleaved fibrinogen to form fibrin clots significantly stronger than those formed by the venoms (*p* < 0.0001 for *T*. *flavomaculatus*; *p* = 0.0007 for *T*. *mcgregori*).

**Figure 6:**
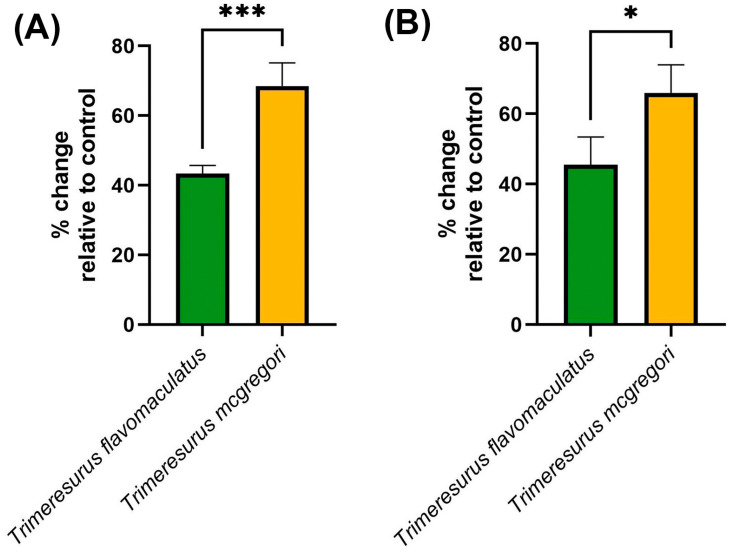
Clotting factor inhibition by the venom samples. Shown here are % change in fibrinogen clotting time relative to the negative control, where 0 means no inhibition, positive values mean inhibition of the respective clotting factor, and negative values imply enhancement of clotting factor activity. (**A**), fIXa; (**B**), fXa. Data are presented as mean ± SD, *n* = 4; asterisks indicate significant differences between the clotting factor inhibition values: *** = *p* < 0.001; * = *p* < 0.05.

Clotting factor inhibition assays (Figure 6) were consistent with the trends observed in both the coagulation analyses and thromboelastography. The key hemostatic factors fIXa and fXa were inhibited by the venom samples, with *T*. *mcgregori* exhibiting more potent inhibition than *T*. *flavomaculatus* in both clotting factors (fIXa: *p* = 0.0004; fXa: *p* = 0.0108).

## 3. Discussion

Our study provided information on the potential coagulotoxic effects of envenomation by two species of Philippine pit vipers. Coagulation analyses showed that both *T*. *flavomaculatus* and *T*. *mcgregori* were able to clot plasma and fibrinogen (Figure 1), and subsequent thromboelastography revealed that the clots formed were substantially weaker than those formed by thrombin (Figure 4 and Figure 5). These results are consistent with observed pseudo-procoagulant (also known as: thrombin-like) activities of some viper venoms, wherein the fibrinogenolytic effects lead to a net anticoagulant state as the formed fibrin clots are weak, unstable, and quickly broken down, depleting fibrinogen levels, thereby contributing to hemorrhagic shock effects [45,46,47,48]. The net anticoagulant effect would be potentiated due to the parallel inhibition of clotting enzymes fIXa and fXa (Figure 6). The monovalent and polyvalent antivenoms used in the study were found to be effective in cross-neutralizing venom toxins, with the Hemato Polyvalent Antivenom exhibiting higher potency than its monovalent counterpart across all samples (Figure 2 and Figure 3; Table 1).

While the coagulotoxicity of *T*. *flavomaculatus* in the present study reflected venom activity observed in congeneric species [49], the results deviated from those gathered in a previous study from our laboratory using *T*. *flavomaculatus* venom sourced from a commercial supplier [50]. Clotting times measured in the present study were noticeably lower than the previous investigation, and thromboelastography revealed that the current samples were able to clot fibrinogen to some extent, which is opposed to the no clotting observed in the previous work. Moreover, clotting factors IXa and Xa were inhibited to a greater extent in the present study, contributing to the net anticoagulant activity. These observed differences in coagulotoxicity by *T*. *flavomaculatus* suggest that regional variation occurs within this species, but this must be tested in the future through the use of additional locality-specific venoms to consider whether such variation impacts antivenom efficacy.

Antivenom is part of the Model List of Essential Medicines compiled by the World Health Organization and remains the first and best line of defense to prevent irreversible effects upon envenomation [51]. However, antivenom production is labor- and resource-intensive [52], hence why heterologous antivenoms are being tested for cross-neutralization activity [53,54,55,56,57]. As such, the possibility of importing antivenoms to address local shortages can be considered, especially by countries which experience a high prevalence of snakebite [58].

In the Philippines, snakebite treatment relies to a certain extent on the use of crude plant extracts and patients opting to visit “faith healers” because of the inaccessibility of healthcare due to distance and cost [33,34]. Moreover, the Philippine Cobra Antivenom (PCAV) against *Naja philippinensis* remains the only antivenom available despite the high diversity of venomous snakes in the country. PCAV is indicated in cases of envenomation by both *N. philippinensis* and *N. samarensis* as their venom toxins are relatively similar, but the cross-neutralizing potency of PCAV is reduced in *N*. *samarensis* nonetheless [59,60]. This lack of coverage in antivenom is further reflected in the generalized treatment guidelines provided by the government; the Philippines’ Department of Health released an advisory on snakebite treatment that states, “Antivenom is the only effective antidote for snake venom” [61], with no indication of the appropriate antivenoms that must be used. Taken together with a poor epidemiological record of snakebite and inadequate access even to PCAV [62], the burden of snakebites in the Philippines remains underappreciated and must be addressed [18,23,36].

Despite the results of this study showing cross-neutralization of *T*. *flavomaculatus* and *T*. *mcgregori* venoms by both the monovalent and the polyvalent antivenoms, we make no claims that these can permanently replace the development of Philippines-specific pit viper antivenoms. The use of non-specific, out-of-region antivenoms to treat envenomation has met with some success both in vitro and in clinical cases of venomous snakebite [48]. However, it typically suffers from a reduced potency compared to specific antivenom neutralization, even against venoms of similar species [63,64,65,66,67]. In the absence of specific antivenoms, however, our results suggest that the Philippines can benefit from cross-neutralizing antivenoms until specific antivenoms targeting medically important venomous snakes can be developed. Thus, by temporarily importing non-specific antivenoms, the Philippines can be better prepared in mitigating the burden of snakebites.

## 4. Materials and Methods

### 4.1. Venom Preparation

Venom from *T. flavomaculatus* was collected and pooled from adult individuals (*n* = 7) sourced from the Bicol Region and reared by the Avilon Wildlife Conservation Foundation, 9003 GP Sitio Gulod, Rodriguez, Rizal 1860 Philippines. Venom collection was authorized under Wildlife Gratuitous Permit No. R4A-WGP-2023-RIZ-017 by the Department of Environment and Natural Resources (Philippines), and storage at the University of Santo Tomas was approved by the UST Institutional Biosafety Committee. *T*. *mcgregori* venom was obtained from the long-term cryogenic collection of the Adaptive Biotoxicology Lab from pools of two captive born adult male and two adult females. All venoms used in the study were lyophilized and stored at −80 °C prior to preparation.

Venoms were reconstituted to 1 mg/mL stock solutions (1:1 double deionized water (ddH_2_O):glycerol) monitored at 280 nm wavelength using a Thermo Fisher Scientific™ NanoDrop 2000 UV–Vis spectrophotometer (Waltham, MA, USA). Venom stocks were stored at −20 °C during experimentation.

### 4.2. Antivenom Preparation

Antivenoms sourced from the Queen Saovabha Memorial Institute, Thai Red Cross Society, Bangkok, Thailand were used to test for cross-neutralization. They were the Hemato Polyvalent Antivenom (Lot No. HP00323; expiry date 13 June 2028) raised against viperids with known hemotoxic venom (*Daboia siamensis*, *Calloselasma rhodostoma*, and *Trimeresurus albolabris*), and the monovalent Green Tree Pit Viper Antivenom (Lot No. TA00119; expiry date 15 January 2024) raised against *T. albolabris*. The antivenoms were dissolved in 10 mL of sterile ddH_2_O according to the manufacturer’s protocol and centrifuged at 14,000× *g* for 10 min at 4 °C. The supernatants of each antivenom were prepared in Owren Koller (OK) buffer to generate 5% antivenom solutions. These solutions were then stored at −20 °C until required in cross-neutralization assays of *T*. *flavomaculatus* and *T*. *mcgregori* venoms.

### 4.3. Plasma and Fibrinogen Preparation

Handling and use of human plasma was authorized by the University of Queensland Biosafety Approval # ICB134BSBS2015 and Human Ethics Approval # 2016000256. Human platelet-poor plasma (3.2% citrated) was supplied by the Australian Red Cross (44 Musk Street, Kelvin Grove QLD 4059 Australia) under research approval # 16-04QLD-10. Plasma was aliquoted into 1.5 mL tubes within a biosafety cabinet to prevent contamination and stored at −80 °C until needed.

Fibrinogen (Sigma Aldrich, St. Louis, MO, USA) was prepared to 4 mg/mL by dissolving 100 mg of fibrinogen with Owren Koller (OK) buffer to a volume of 25 mL. The solution was vortexed until the fibrinogen completely dissolved; after which, it was aliquoted into 1.5 mL tubes. Aliquots were flash-frozen with liquid nitrogen and stored at −80 °C until required.

### 4.4. Coagulation Analysis and Antivenom Cross-Neutralization

Coagulation assays were performed using a Stago^®^ STA R Max coagulation analyser running Stago Analyser software v0.00.04 (Stago, Asnières sur Seine, France) following previously described methods with some modifications [50,68,69]. Plasma or fibrinogen was warmed to 37 °C in a water bath for 5 min. Venom from the ddH_2_O:glycerol stock was diluted to a 100 µg/mL working solution with OK buffer. From this venom solution, 50 µL was taken and added to 50 µL of 0.025 M CaCl_2_ (Stago cat# 00367), 25 µL OK buffer, and 50 µL phospholipid (Stago cat# 00597). Next, the solution was incubated at 37 °C for 120 s; after which, 75 µL plasma or fibrinogen was added. Clotting time was monitored until the plasma or fibrinogen clotted or the machine’s maximum monitoring time of 999 s was reached. These were performed to generate initial clotting time data with venom concentration at 20 µg/mL and to obtain dose–response curves with 9 venom concentrations (40 µg/mL, 20 µg/mL, 10 µg/mL, 4 µg/mL, 1.67 µg/mL, 0.67 µg/mL, 0.25 µg/mL, 0.125 µg/mL, and 0.05 µg/mL) in plasma or fibrinogen. Negative controls were run by substituting the venom sample with 50 µL of 1:1 ddH_2_O:glycerol, while positive controls were run by using 50 µL kaolin (Stago cat# 00597) in place of venom.

Antivenom cross-neutralization tests were conducted by replacing the 25 µL OK buffer in the coagulation tests with 25 µL of the Hemato Polyvalent Antivenom or the monovalent antivenom, leading to a final antivenom concentration of 0.5% per cuvette. Clotting time was monitored in a similar manner to the coagulation analysis and dose–response curves with the same 9 venom concentrations were generated to measure whether the antivenoms were able to neutralize venom toxins, as exhibited by significant effects on clotting time.

### 4.5. Clotting Factor Inhibition

The venoms were tested on the STA R Max coagulation analyzer for their ability to inhibit the clotting factors fIXa and fXa following previously validated methods [70,71,72]. Into each cuvette, 25 µL of venom (0.2 µg/mL), 50 µL CaCl_2_, 25 µL OK buffer, 50 µL phospholipid, and 25 µL of fIXa (Prolytix cat# HCIXA-0050) or fXa (Stago cat# 00811) were added, incubated for 2 min at 37 °C, and then 75 µL of human plasma was added and the clotting time was measured. Negative controls were run by substituting the venom sample with 50 µL of 1:1 ddH_2_O:glycerol.

### 4.6. Thromboelastography

Two TEG^®^ 5000 Thromboelastograph^®^ (Haemonetics Australia Pty Ltd., Macquarie Park, NSW, Australia) hemostasis analyzer systems were used to measure venom effects on clotting strength in either plasma or fibrinogen. Plain cups and pins (Haemonetics cat# 6211) were placed into each channel of the analyzers and warmed to 37 °C. Reagents for the reaction were then pipetted into each cup according to our previously validated protocol [46,73]: 72 µL CaCl_2_ (25 mM stock solution Stago cat# 00367) and 72 µL phospholipid (Stago cat# 00597) dissolved in Owren Koller (OK) buffer (Stago cat# 00360); 20 µL OK buffer; and 7 µL of either the control reagent (negative control: 1:1 ddH_2_O:glycerol; positive thrombin control: thrombin) or a 1 mg/mL venom sample. Prior to starting the reaction, 189 µL of either plasma or fibrinogen, which were thawed at 37 °C for 5 min in a water bath, were pipetted into each cup. The 360 µL solutions were then pipette mixed, and the analyzers were run for 30 min. To minimize residual interactions in the solutions, the time between the pipetting of the plasma or fibrinogen and the start of the reaction was kept to a maximum of 10 s. All reactions were performed in triplicate (*n* = 3).

### 4.7. Data Analysis and Visualization

A one-way ANOVA was performed to compute the overall statistical differences in the venom clotting analyses, with Dunnett’s multiple comparison test performed as a post hoc analysis to determine whether the venom-induced clotting times were significantly different from the negative control. The area under the curve (AUC) was computed for the venom dilution curves and the resulting AUC values were used to compute for the percentage shift in clotting time between the negative control and the antivenoms. Percentage shift was calculated by the following formula:

$$\%shift=\left(\frac{AUC\;of\;venom+antivenom}{AUC\;of\;venom}-1\right)\times 100$$

where a value above 0 indicates venom neutralization by the antivenom being tested [74]. Overall differences among the AUC values of each venom and antivenom pair were computed through a one-way ANOVA then compared to each other using the Tukey–Kramer multiple comparison test. Statistical differences between the venom and antivenom pairs of each venom sample were visualized. All statistical analyses and graphical visualizations were conducted on GraphPad Prism^®^ 10.4.1 (GraphPad Software, Boston, MA, USA).

Thromboelastography tracings were exported from the TEG 5000 Analytical Software (Haemonetics cat# 07-031). Figures containing these tracings were produced in Adobe Photoshop 26.2.0 (Adobe, Inc., San Jose, CA, USA).

## Figures and Tables

**Table 1:** Antivenom cross-neutralization of venom samples across multiple concentrations. Shown here are *p*-values from the Tukey–Kramer multiple comparison test between the negative control (venom + fibrinogen) and the venom sample in the presence of the corresponding antivenom. The antivenoms were also compared with each other to find which one provided a higher level of cross-neutralization in each concentration of venom. Concentrations are from the 9-point dilution curves. **N/A:** comparison where the maximum monitoring time of 999 s was reached in both groups being compared. **MAv:** Green Tree Pit Viper Antivenom; **HPAv:** Hemato Polyvalent Antivenom. *: MAv provided significantly higher cross-neutralization. **^**: HPAv provided significantly higher cross-neutralization.

Venom Concentration	*Trimeresurus flavomaculatus*			*Trimeresurus mcgregori*		
	MAv	HPAv	MAv vs. HPAv	MAv	HPAv	MAv vs. HPAv
**40.00 µg/mL**	0.0067	0.0282	0.0039 *	0.1455	0.2252	0.0527
**20.00 µg/mL**	0.0128	0.0193	0.1669	0.6678	0.1997	0.5933
**10.00 µg/mL**	0.0057	0.0160	0.0903	0.2804	0.0189	0.0470 *
**4.00 µg/mL**	<0.0001	0.0043	0.0095 ^	0.0827	0.0606	0.0825
**1.67 µg/mL**	<0.0001	<0.0001	N/A	0.0036	0.0036	N/A
**0.67 µg/mL**	0.2391	0.2391	N/A	N/A	N/A	N/A
**0.25 µg/mL**	N/A	N/A	N/A	N/A	N/A	N/A
**0.125 µg/mL**	N/A	N/A	N/A	N/A	N/A	N/A
**0.05 µg/mL**	N/A	N/A	N/A	N/A	N/A	N/A

## Data Availability

The original contributions presented in this study are included in the article. Further inquiries can be directed to the corresponding author.
